# Value of nutritional indices in predicting survival free from pump replacement and driveline infections in centrifugal left ventricular assist devices

**DOI:** 10.1016/j.xjon.2024.03.017

**Published:** 2024-04-09

**Authors:** Fabian Jimenez Contreras, Bret L. Pinsker, Jason N. Katz, Stuart D. Russell, Jacob Schroder, Benjamin Bryner, Alexander H. Gunn, Krunal Amin, Carmelo Milano

**Affiliations:** a Division of Cardiovascular and Thoracic Surgery, Department of Surgery, Duke University Medical Center, Durham, NC; b Division of Cardiology, Department of Medicine, Duke University Medical Center, Durham, NC

**Keywords:** nutrition, left ventricular assist devices

## Abstract

**Objective:**

There is a paucity of data assessing the impact of nutritional status on outcomes in patients supported with the HeartMate 3 (HM3) left ventricular assist device (LVAD).

**Methods:**

Patients ≥18 years of age who underwent HM3 LVAD implantation between 2015 and 2020 were identified from a single tertiary care center. The primary outcome assessed was death or device replacement. A secondary outcome of driveline infection was also evaluated. Kaplan-Meier survival analysis and a multivariate Cox-proportional hazards model were used to identify predictors of outcome.

**Results:**

Of the 289 patients identified, 94 (33%) experienced a primary outcome and 96 (33%) a secondary outcome during a median follow-up time of 2.3 years. Independent predictors of the primary outcome included peripheral vascular disease (hazard ratio [HR], 3.40; 95% confidence interval [CI], 1.66-6.97, *P* < .01), diabetes mellitus (HR, 0.46; 95% CI, 0.27-0.80, *P* < .01), body mass index ≥40 kg/m^2^ (HR, 2.63 per 1 kg/m^2^ increase; 95% CI, 1.22-5.70, *P* < .05), preoperative creatinine level (HR, 1.86 per 1 mg/dL increase; 95% CI, 1.31-2.65, *P* < .01), and preoperative prognostic nutritional index (PNI) score (HR, 0.88 per 1-point increase; 95% CI, 0.81-0.96, *P* < .01). Independent predictors of driveline infection included age at the time of implantation (HR, 0.97; 95% CI, 0.96-0.99, *P* < .01) and diabetes mellitus (HR, 1.79; 95% CI, 1.17-2.73, *P* < .01).

**Conclusions:**

Preoperative PNI scores may independently predict mortality and the need for device replacement in patients with HM3 LVAD. Routine use of the PNI score during preoperative evaluation and, when possible, supplementation to PNI >33, may be of value in this population.

## Graphical Abstract

**Figure:**
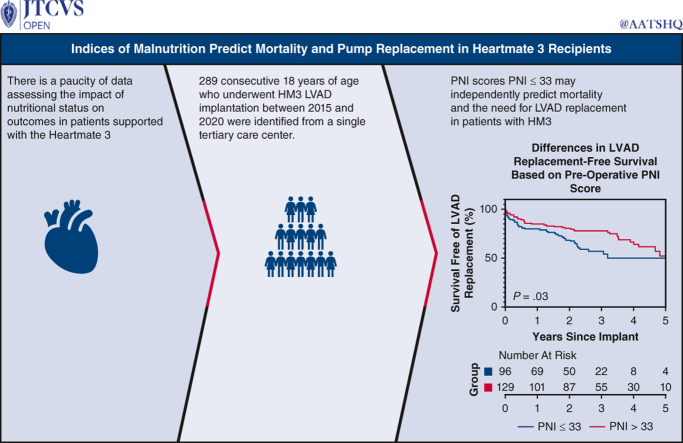

**Figure:**
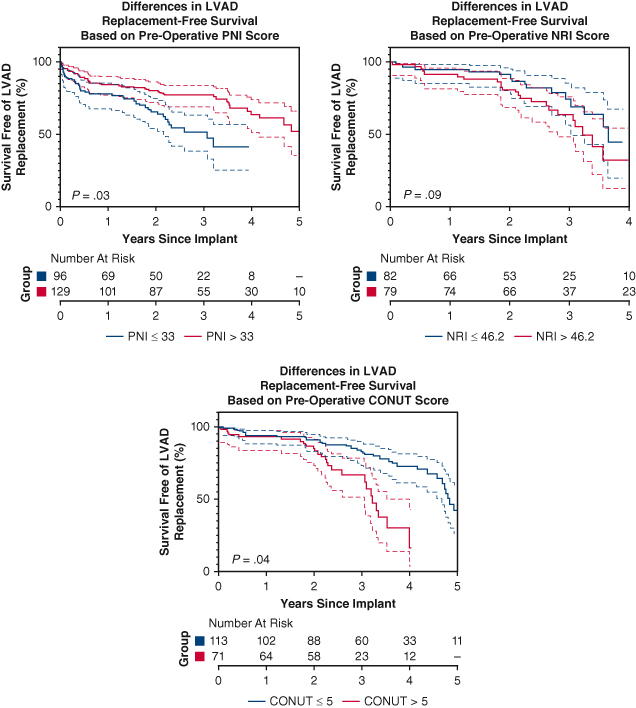
Survival analysis of death or explant based on preoperative PNI, NRI, and CONUT scores.

**Central Message**

Preoperative PNI scores may independently predict mortality and the need for device replacement in patients with HM3 LVAD.

**Perspective**

There is a paucity of data assessing the effect of nutritional status on outcomes in patients supported with the HeartMate 3. The current study evaluated many commonly used nutritional indexes. Routine use of the PNI score during preoperative evaluation and, when possible, supplementation to PNI >33, may be of value in this population.

Left ventricular assist devices (LVADs) are an important treatment for end-stage heart failure, with more than 25,000 patients having received a durable LVAD over the past decade.^1^ Given the limited supply of cardiac allografts and the increasing number of patients developing advanced heart failure, the need for LVADs may increase further.^1^ Currently, the HeartMate 3 (HM3; Abbott Cardiovascular) is the only available third-generation LVAD. It is fully magnetically levitated and has shown lower rates of stroke and need for replacement than its predecessor, the HeartMate II (Abbott Cardiovascular).^1^

Although poor nutritional status is a well-established risk factor for adverse outcomes in advanced heart failure, the prognostic value of nutritional markers in patients undergoing LVAD implantation is unclear. The International Society for Heart and Lung Transplantation 2023 Guidelines currently recommend the routine assessment of a patient's nutritional state before implantation of durable mechanical circulatory support and, when possible and necessary, supplementation.^2^ However, these recommendations are ambiguous and offer no specific preimplantation targets toward which providers and patients can work. This is predominantly the result of limited data assessing the prognostic value of nutritional markers in this population.

Although some studies have demonstrated prognostic value with measurement of albumin, prealbumin, total cholesterol, and total lymphocyte counts, other studies have failed to demonstrate similar results.^3^, ^4^, ^5^, ^6^, ^7^, ^8^ These markers have also been incorporated into multivariable scoring systems, some of which may be helpful in risk-stratifying patients undergoing cardiac surgery. For example, the prognostic nutritional index (PNI) score—originally developed in prognosticating patients undergoing gastrointestinal surgery—was found to be associated with postoperative mortality in a cohort of patients undergoing LVAD implantation.^9^ Separately, the nutritional risk index (NRI) score, first developed by the Veterans’ Affairs Total Parenteral Nutrition Cooperative Study, demonstrated a correlation with mortality in a cohort of patients with chronic heart failure, as well as device infections in a separate cohort.^10^^,^^11^ Finally, the Controlling Nutritional Status (CONUT) score, which was originally created in 2005 to help identify undernourished patients in the hospitalized setting, may also help prognosticate patients undergoing LVAD implantation, as it has previously been shown to be associated with mortality and infection in hospitalized patients with congestive heart failure.^3^^,^^12^ Although all of these analyses suggest potential value among certain nutritional markers/scoring systems, they have unfortunately consisted of small sample sizes or cohorts of patients with predominantly older LVAD models. This limits their generalizability, especially among patients with newer generation HM3 LVADs.

Given that the HM3 LVAD is the standard of care for the foreseeable future, assessing predictors of adverse outcomes in this population is important. This study aims to characterize what factors, including markers of nutritional status, may help predict survival free from LVAD replacement or driveline infection in patients with HM3 LVAD.

## Patients and Methods

### Ethics Statement

Approval for this project was given by the institution's institutional review board on January 30, 2019 (Pro00101472). The need for individual patient informed consent was waived. Data were deidentified to protect patient privacy.

### Patient Selection and Data Source

The study group was composed of all consecutive patients ≥18 years of age who underwent HM3 LVAD implantation between 2015 and 2020 at a single tertiary care center. All variables were gathered from the electronic medical record, corresponding to the hospitalization at the time of HM3 LVAD implantation. Demographic variables included age at the time of implantation, sex, and race. Medical history was reviewed, and pertinent variables were collected, including a history of hypertension, chronic kidney disease, tobacco use, peripheral vascular disease (PVD) (as defined by ankle brachial index <0.9, angiography with vessel narrowing ≥50%, or a history of revascularization), and diabetes mellitus (DM). Clinical variables assessed included body mass index (BMI), preoperative intra-aortic balloon pump use, and preoperative extracorporeal membrane oxygenation use. Preoperative creatinine, total bilirubin, albumin, prealbumin, and transferrin, total lymphocyte count, and total cholesterol were also gathered closest in time to but no later than 1 month before HM3 LVAD implantation. These variables were also used to calculate preoperative NRI scores ($1.519*\;\text{Preoperative}\;\text{albumin}\;\left(g/L\right)+41.7*\;\frac{\text{Preoperative}\;\text{weight}\;\left(\text{kg}\right)}{\text{Baseline}\;\text{weight}\;\left(\text{kg}\right)}$), PNI scores ($10*\text{Preoperative}\;\text{albumin}\;\left(g/L\right)+0.005*{\;\text{Preoperative}\;\text{lymphocyte}\;\text{count}\;(*10}^{9}/L)$), and CONUT scores (Table 1).^3^ Reference ranges for NRI, PNI, and CONUT scores are displayed in Table 2.

**Table 1:** Components of CONUT score

	Parameter		
	Preoperative albumin, g/dL	Preoperative total lymphocytes, ∗10^9^/L	Preoperative total cholesterol, mg/dL
Score			
0	≥3.50	≥1600	≥180
1	–	1200-1599	140-179
2	3.00-3.49	800-1199	100-139
3	–	<800	<100
4	2.50-2.99	–	–
6	<2.50	–	–

*CONUT*, Controlling Nutritional Status.

**Table 2:** Reference ranges for various NRI, PNI, and CONUT scores

Nutritional index	Normal	Mild malnutrition	Moderate malnutrition	Severe malnutrition
NRI score	≥100	97.5-100	83.5-97.4	<83.5
PNI score	≥38	N/a	35-37.9	<35
CONUT score	0-1	2-4	5-8	9-12

*NRI*, Nutritional risk index; *PNI*, prognostic nutritional index; *CONUT*, Controlling Nutritional Status.

### Outcomes

The primary outcome was death or HM3 LVAD replacement, and the secondary outcome LVAD driveline infection. Patients who underwent HM3 LVAD replacement as the result of hardware malfunction were not included in the analysis. Driveline infection was defined as a positive culture from the skin/tissue surrounding the driveline necessitating antimicrobial therapy, debridement, and/or device replacement. Patients who were transplanted while on LVAD support were censored from the analysis at the time of transplant. Follow-up time was defined as the time from HM3 LVAD implantation to the development of an outcome or the date of last follow-up. Outcomes were determined via the review of electronic medical records.

### Statistical Analysis

Depending on the distribution of the data, continuous variables are displayed as either a mean ± standard deviation or a median with an interquartile range. Categorical variables are displayed as numbers and percentages. Baseline demographic and clinical characteristics were compared using either an unpaired *t*-test or Mann-Whitney *U* test for continuous variables and a χ^2^ or Fisher exact test for categorical variables.

Kaplan-Meier survival analysis with log-rank testing was performed using GraphPad Prism 9 to compare differences in outcome-free survival among patients based on preoperative PNI, NRI, and CONUT scores. Median preoperative PNI, NRI, and CONUT scores of the cohort were used as cutoffs in the Kaplan-Meier analysis.

Factors were assessed for an association with outcome development using a univariable Cox proportional hazards regression. Independent association with outcome development was then determined using a multivariable, backward, stepwise regression model (*P*-removal 0.10). The final model selection was based on goodness-of-fit tests (Akaike information criterion and Bayesian information criterion). Collinearity was assessed for and, if present, taken into account before final model selection.

## Results

In total, 289 consecutive patients ≥18 years of age who underwent HM3 LVAD implantation were identified. The median age at the time of HM3 LVAD implantation was 62.2 years. Demographic, clinical, and laboratory characteristics of the cohort are displayed in Table 3.

**Table 3:** Patient characteristics

Variable	Total patients (n = 289)	Death or LVAD replacement		*P* value
		No (n = 195)	Yes (n = 94)	
Median age at implant, y (IQR)	62.2 (50.0-69.0)	60.0 (49.0-68.0)	65.1 (51.8-70.0)	.02
Median length of follow-up, y (IQR)	2.3 (1.2-3.5)	2.7 (2.0-3.9)	1.2 (0.3-2.2)	<.01
Female, n (%)	58 (20%)	38 (20%)	20 (21%)	.72
Black/African American	128 (44%)	90 (46%)	38 (40%)	.36
Height, cm	175.0 (170.0-183.0)	177.0 (170.5-183.0)	175.0 (166.5-183.0)	.41
Weight, kg	93.6 (77.3-112.3)	93.6 (77.9-112.0)	94.9 (75.5-114.6)	.93
BMI, kg/m^2^	29.6 (25.3-35.8)	29.5 (25.1-35.9)	30.2 (25.8-35.7)	.58
BSA, m^2^	2.1 (1.9-2.4)	2.1 (1.9-2.4)	2.2 (1.9-2.3)	.91
Hypertension	196 (68%)	136 (70%)	61 (64%)	.31
Smoker	194 (67%)	135 (69%)	59 (63%)	.27
Peripheral vascular disease	21 (7%)	10 (5%)	11 (12%)	.04
Diabetes mellitus	125 (43%)	86 (44%)	39 (41%)	.67
Preoperative aortic insufficiency	30 (10%)	18 (9%)	12 (13%)	.36
Preoperative IABP	93 (32%)	68 (35%)	25 (27%)	.16
Preoperative ECMO	21 (7%)	13 (7%)	8 (9%)	.57
Previous LVAD	51 (18%)	33 (17%)	19 (20%)	.50
INTERMACS profile	2.7 (1.8-3.7)	2.7 (2.5-2.8)	2.8 (2.7-3.0)	.21
Markers of nutrition				
Preoperative creatinine, mg/dL	1.4 (1.1-1.8)	1.3 (1.0-1.7)	1.5 (1.1-1.9)	.03
Preoperative total bilirubin, mg/dL	1.2 (0.9-1.9)	1.3 (0.9-1.9)	1.1 (0.8-1.7)	.03
Preoperative Albumin, g/dL	3.3 (3.0-3.7)	3.3 (3.0-3.7)	3.2 (2.9-3.6)	.13
Preoperative prealbumin, mg/dL	18.0 (12.9-22.8)	19.3 (14.0-23.2)	13.4 (10.4-19.7)	.02
Preoperative transferrin, mg/dL	215.8 (156.0-270.3)	211.0 (138.7-273.0)	219.0 (202.5-245.0)	.47
Preoperative total lymphocytes, ∗10^9^/L	1.2 (0.9-1.7)	1.3 (0.9-1.7)	1.2 (0.9-1.6)	.29
Preoperative total cholesterol, mg/dL	121.5 (97.0-152.0)	126.0 (94.0-155.0)	119.0 (100.0-149.0)	.90
PNI score	33.0 (30.0-37.0)	34.0 (31.0-37.0)	32.0 (28.0-36.0)	.03
NRI score	46.2 (43.8-47.9)	46.3 (44.1-47.6)	46.0 (43.4-47.6)	.91
CONUT score	5.0 (3.0-6.0)	5.0 (3.0-6.0)	5.0 (4.0-7.0)	.11

*LVAD*, Left ventricular assist device; *IQR*, interquartile range; *BMI*, body mass index; *BSA*, body surface area; *IABP*, intra-aortic balloon pump; *ECMO*, extracorporeal membrane oxygenation; *INTERMACS*, Interagency Registry for Mechanically Assisted Circulatory Support; *PNI*, prognostic nutritional index; *NRI*, nutritional risk index; *CONUT*, Controlling Nutritional Status.

Over a median follow-up time of 2.3 years, 94 patients (33%) experienced a primary outcome, and 96 (33%) experienced a secondary outcome. Of the 94 patients who experienced a primary outcome, 12 required HM3 LVAD replacement, all for device infection, and 83 experienced death. One patient experienced death after LVAD replacement. Thirty-six patients (12%) underwent transplant and were censored from the analysis at the time of transplant. Outcomes occurred between 6 days and 6.8 years after HM3 LVAD implantation.

Compared with patients who did not experience the primary outcome, patients who died or underwent device replacement were older at the time of initial HM3 implantation and more likely to have a history of PVD, elevated preoperative creatinine levels, depressed preoperative bilirubin levels, and depressed preoperative prealbumin levels. Patients who experienced the primary outcome also had lower PNI scores than their counterparts (Table 3).

**Figure 1:**
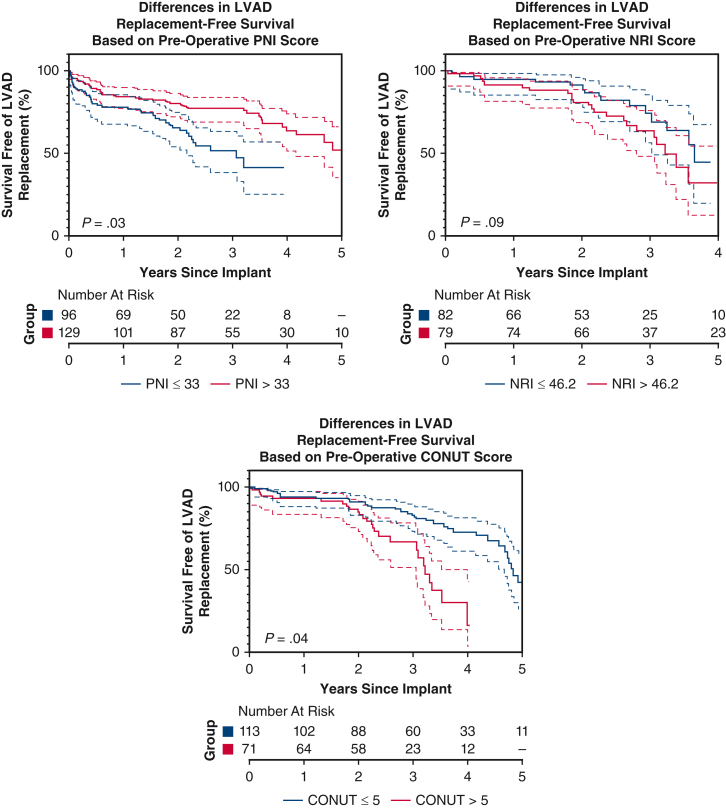
Kaplan-Meier survival analysis of primary outcome (death or device explant) based on preoperative PNI, NRI, and CONUT scores. 95% confidence intervals shown. *P* values are derived from log-rank test. *PNI*, Prognostic nutritional index; *NRI*, nutritional risk index; *CONUT*, Controlling Nutritional Status; *LVAD*, left ventricular assist device.

Kaplan-Meier survival analysis is displayed in Figure 1. Patients with PNI ≤33 were more likely to experience death or pump replacement compared with patients with PNI >33 (*P* = .03). Death and pump replacement were also more common among patients with CONUT ≤5 than patients with CONUT >5 (*P* = .04). Survival free from LVAD replacement was similar among patients with NRI ≤46.2 and NRI>46.2 (*P* = .09).

**Figure 2:**
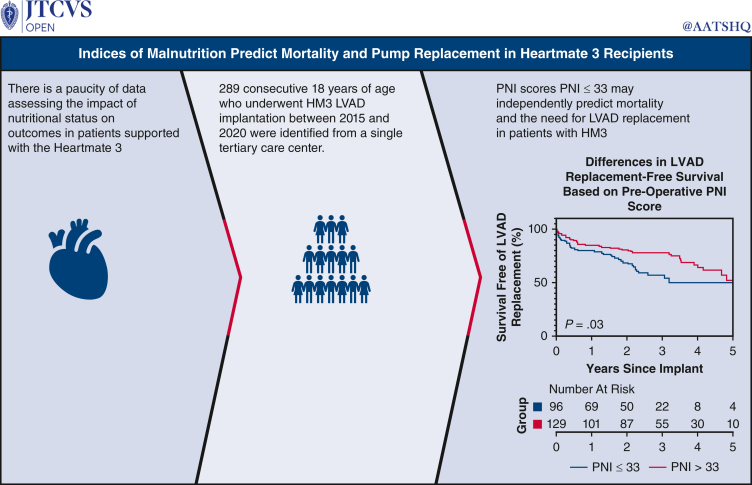
Graphical abstract.

Several metrics were found to be predictive of survival free from LVAD replacement on univariable Cox-proportional hazards regression analysis (Table 4). Metrics that were associated with an increased risk for the primary outcome included a history of PVD (HR, 2.50; 95% CI, 1.33-4.72, *P* < .01), the use of extracorporeal membrane oxygenation before implantation (HR, 2.20; 95% CI, 1.06-4.59, *P* = .04), and preoperative creatinine levels (HR, 1.50 per 1 mg/dL increase; 95% CI, 1.14-1.97, *P* < .01). Patients with elevated albumin levels at the time of LVAD implantation were less likely to experience outcome development (HR, 0.56 per 1 g/dL increase; 95% CI, 0.38-0.83, *P* < .01). Nutrition scores that were predictive of the primary outcome included PNI score (HR, 0.95 per 1 point increase; 95% CI, 0.91-0.98, *P* < .01) and CONUT score (HR, 1.12 per 1 point increase; 95% CI, 1.01-1.24, *P* < .05). Covariates associated with driveline infection on univariate analysis are also displayed in Table 4 and included 30 ≤BMI (kg/m^2^) <40 (HR, 1.58; 95% CI, 1.02-2.45, *P* < .05), age (HR, 0.98 per 1 year increase, 95% CI, 0.96-0.99, *P* < .01), and DM (HR, 1.56; 95% CI, 1.04-2.33, *P* = .03).

**Table 4:** Univariate Cox-proportional hazard ratio analysis

	Survival free from device replacement			Driveline infection		
Variable	Hazard ratio	95% CI	*P* value	Hazard ratio	95% CI	*P* value
BMI (kg/m^2^) <30	–	–	–	–	–	–
30 ≤ BMI (kg/m^2^) <40	1.19	0.76-1.85	.455	1.58	1.02-2.45	.040
BMI (kg/m^2^) ≥40	1.11	0.61-2.03	.725	1.39	0.74-2.60	.307
Age (y) at implant per 1-year increase	1.01	0.99-1.03	.266	0.98	0.96-0.99	.001
Female, %	0.94	0.57-1.55	.801	1.54	0.97-2.45	.069
Black/African American, %	0.93	0.62-1.41	.733	1.33	0.88-1.99	.173
Hypertension, %	0.82	0.54-1.25	.353	1.44	0.92-2.27	.115
Smoker, %	0.82	0.54-1.25	.354	1.02	0.66-1.57	.932
Peripheral vascular disease, %	2.50	1.33-4.72	.005	0.44	0.14-1.40	.164
Diabetes mellitus, %	0.87	0.58-1.31	.503	1.56	1.04-2.33	.032
Preoperative aortic insufficiency, %	1.33	0.73-2.44	.356	0.91	0.46-1.82	.914
Preoperative IABP, %	0.89	0.57-1.42	.633	1.00	0.64-1.54	.995
Preoperative ECMO, %	2.20	1.06-4.59	.035	0.88	0.36-2.18	.781
Previous LVAD, %	1.29	0.78-2.15	.319	0.99	0.58-1.70	.964
INTERMACS profile	1.06	0.85-1.32	.599	1.03	0.83-1.27	.801
Preoperative creatinine per 1 mg/dL increase	1.50	1.14-1.97	.004	0.83	0.58-1.18	.298
Preoperative total bilirubin per 1 mg/dL increase	1.02	0.92-1.13	.750	1.01	0.92-1.11	.796
Preoperative albumin per 1 g/dL increase	0.56	0.38-0.83	.004	0.86	0.58-1.27	.454
Preoperative prealbumin per 1 mg/dL increase	0.91	0.82-1.00	.050	0.90	0.78-1.05	.189
Preoperative transferrin per 1 mg/dL increase	1.00	0.99-1.01	.696	1.00	0.99-1.01	.497
Preoperative total lymphocytes per 1∗10^9^/L increase	0.80	0.55-1.15	.228	1.14	0.82-1.59	.429
Preoperative total cholesterol per 1 mg/dL increase	1.00	0.99-1.00	.687	1.00	1.00-1.01	.463
PNI score per 1-point increase	0.95	0.91-0.98	.002	0.99	0.95-1.03	.523
NRI score per 1-point increase	0.99	0.93-1.06	.801	1.01	0.95-1.07	.840
CONUT score per 1-point increase	1.12	1.01-1.24	.032	1.03	0.93-1.14	.593

*CI*, Confidence interval; *BMI*, body mass index; *IABP*, intra-aortic balloon pump; *ECMO*, extracorporeal membrane oxygenation; *LVAD*, left ventricular assist device; *INTERMACS*, Interagency Registry for Mechanically Assisted Circulatory Support; *PNI*, prognostic nutritional index; *NRI*, nutritional risk index; *CONUT*, Controlling Nutritional Status.

Independent predictors of survival free from device replacement are displayed in Table 5 and included a history of PVD (HR, 3.40, 95% CI, 1.66-6.97, *P* < .01), preoperative creatinine levels (HR, 1.86 per 1 mg/dL increase; 95% CI, 1.31-2.65, *P* < .01), DM (HR, 0.46; 95% CI, 0.27-0.80, *P* < .01), preoperative PNI score (HR, 0.88 per 1 point increase; 95% CI, 0.81-0.96, *P* < .01), and BMI (kg/m^2^) ≥40 (HR, 2.63 per 1 kg/m^2^ increase, 95% CI, 1.22-5.70, *P* < .05). A separate regression analysis identified age (HR, 0.97; 95% CI, 0.96-0.99, *P* < .01) and DM (HR, 1.79; 95% CI, 1.17-2.73, *P* < .01) as independent predictors of driveline infection (Table 6).

**Table 5:** Multivariate Cox-proportional hazards ratio analysis for death or device replacement

Variable	Hazard ratio	95% CI	*P* value
Age (y) at implant per 1-year increase	1.02	1.00-1.05	.081
Peripheral vascular disease	3.40	1.66-6.97	<.001
Diabetes mellitus	0.46	0.27-0.80	.005
BMI (kg/m^2^) <30	–	–	–
30 ≤ BMI (kg/m^2^) <40	1.59	0.87-2.91	.133
BMI (kg/m^2^) ≥40	2.63	1.22-5.70	.014
Preoperative creatinine per 1 mg/dL increase	1.86	1.31-2.65	<.001
PNI score per 1-point increase	0.88	0.81-0.96	.002
CONUT score per 1-point increase	0.95	0.81-1.13	.570

*CI*, Confidence interval; *BMI*, body mass index; *PNI*, prognostic nutritional index; *CONUT*, Controlling Nutritional Status.

**Table 6:** Multivariate Cox-proportional hazards ratio analysis for driveline infection

Variable	Hazard ratio	95% CI	*P* value
Age (y) at implant per 1-year increase	0.97	0.96-0.99	.001
Diabetes mellitus	1.79	1.17-2.73	.007
Peripheral vascular disease	0.41	0.13-1.31	.133
BMI (kg/m^2^) <30	–	–	–
30 ≤ BMI (kg/m^2^) <40	1.27	0.81-2.00	.301
BMI (kg/m^2^) ≥40	0.97	0.51-1.87	.929

*CI*, Confidence interval; *BMI*, body mass index.

## Discussion

The prognostic value of nutritional markers for patients supported with LVAD is unclear. Although some studies suggest preoperative albumin levels, PNI, and NRI scores may better predict morbidity and mortality after LVAD implantation, other studies have failed to demonstrate this association.^5^, ^6^, ^7^^,^^13^ As such, no strong, uniform method of preimplantation nutritional assessment exists for patients undergoing LVAD implantation. Our study consists of one of the largest single-institution cohorts of patients with the HM3 LVAD and identifies a variety of nutritional indices that may help predict survival free from pump replacement and driveline infections. This is especially valuable, given the lack of data assessing prognostic variables in this population, in part resulting from the relative novelty of the HM3 device.

Our results suggest that preoperative PNI scores may help predict survival free from pump replacement in patients undergoing HM3 LVAD implantation. This aligns with previous studies that have assessed the prognostic value of preoperative PNI scores in cohorts with older LVAD models, as well as patients hospitalized with acute heart failure.^9^ Although albumin and total lymphocytes levels (both of which are used to calculate the PNI score) are likely influenced by a number of pathophysiologic mechanisms, they are generally considered to be strong indicators of overall nutritional status.^14^ Depressed levels may indicate a mismatch in anabolic and catabolic metabolism that ultimately promotes maladaptive energy consumption, persistent inflammation and, over time, malnutrition.^15^ In patients with advanced heart failure who are already in a tenuous clinical state, such processes may confer added risk for morbidity and mortality.

These findings augment the recent 2023 International Society for Heart and Lung Transplantation Guidelines for Mechanical Circulatory Support, as they display a decreased likelihood of death and pump replacement among patients with PNI >33. Notably, a PNI value of 33 still falls under the category of “severe” malnutrition, and (for many) represents a realistic, achievable target in patients with advanced heart failure and chronic malnutrition. As such, our results suggest potential value with the routine assessment of preoperative PNI scores in this population and, when possible, supplementation to PNI >33 before LVAD implantation.

The NRI and CONUT scores were not significantly predictive of death or device replacement in our analysis. Unlike the NRI and CONUT scores, the PNI score may be more predictive of death and pump replacement, given the inherent emphasis it places on preoperative albumin levels, which have previously been associated with adverse outcomes in this population.^6^ Markers such as total cholesterol and preoperative weight, which are factored into the NRI and CONUT scores, have not traditionally been associated with adverse outcomes in this population. Future work should be aimed in the direction of developing other scoring systems that continue to place an emphasis on preoperative albumin levels, while also incorporating additional indices of nutrition.

In line with previous studies, our results suggest that a BMI ≥40 kg/m^2^ confers an increased risk of death or device replacement after HM3 LVAD implantation.^4^^,^^8^^,^^16^ That said, 30 ≤ BMI (kg/m^2^) < 40 was unassociated with the primary outcome. This may be due to the relative lack of patients who underwent device replacement as the result of pump thrombosis, which has previously been associated with elevated BMI in this population.^1^^,^^17^

In our analysis, elevated preoperative creatinine was independently associated with an increased risk for death or device replacement. Serum creatinine levels are associated with overall muscle mass and thus may be low in patients who are underweight or malnourished.^18^ However, serum creatinine levels are greatly influenced by an individual's overall renal function. Given that renal dysfunction is common in patients with advanced heart failure, preoperative creatinine levels may be more closely associated with overall renal function than nutritional status.

Consistent with previous studies, our results suggest that a history of PVD is associated with an increased risk for LVAD replacement and death.^19^ Similar to other LVAD, the HM3 may exacerbate the harmful effects of PVD by contributing to additional continuous flow-induced endothelial dysfunction. HM3 therapy should therefore be considered carefully in patients with known PAD.

Interestingly, there was a decreased likelihood for development of the primary outcome in patients with a history of DM. This may be because patients were not stratified based on type of DM, hemoglobin A1c levels, insulin dependence, and other aspects indicative of overall “severity” of DM. As such, the presence of DM in our cohort may serve as a multifactorial representation of various aspects of overall nutrition status, both “good” and “bad,” including elevated BMI, serum lipid levels, food security, and more. That said, our findings contrast with those in prior studies that have assessed the relationship between DM and adverse outcomes in patients with LVAD.^20^

Several factors were found to be independently associated with driveline infections in patients receiving HM3 LVAD, including younger age and a history of DM. This is in line with previous studies.^4^^,^^20^^,^^21^ It is possible that younger patients are more likely to experience driveline infections because they may be more active and at an increased risk to experience exit site trauma. Separately, a history of DM may confer an increased risk for driveline infection due to underlying mechanisms that impair both innate and cellular-mediated immune responses.^22^

### Limitations

Nutritional research in the advanced heart failure population is challenging, as these patients suffer from numerous comorbidities, many of which can affect overall nutritional status. The implications of our findings may also be limited by the complex relationship between gut malabsorption and advanced heart failure, as well as the relative urgency of LVAD implantation in profoundly ill and even unstable patients.

Although beneficial in certain ways, the single-institution nature of the study introduces bias and limits the overall generalizability of the results. As a retrospective analysis, we cannot exclude the potential impact of unmeasured confounders on the outcomes of interest. In addition, we did not adjust for hemodynamic variables or illness severity markers.

## Conclusions

PVD, greater preoperative creatinine, and lower preoperative PNI score may independently predict mortality and the need for LVAD replacement in patients with HM3 LVAD, whereas a history of DM and younger age may predict driveline infections. The lack of predictive value of BMI in our analysis suggests that HM3 LVAD implantation in patients with advanced heart failure and elevated BMI may be a safe therapeutic option in this patient group, provided that they are otherwise appropriate surgical candidates. The routine assessment of preoperative PNI score and, when possible, supplementation to PNI >33 before implantation, may be beneficial in patients undergoing HM3 LVAD implantation.

## Uncited Figure

Figure 2.

## Conflict of Interest Statement

Dr Milano discloses a financial relationship with Abbott. Dr Katz receives modest research support from Abbott. All other authors reported no conflicts of interest.

The *Journal* policy requires editors and reviewers to disclose conflicts of interest and to decline handling or reviewing manuscripts for which they may have a conflict of interest. The editors and reviewers of this article have no conflicts of interest.
